# Diatoms and Plants Acyl-CoA:lysophosphatidylcholine Acyltransferases (LPCATs) Exhibit Diverse Substrate Specificity and Biochemical Properties

**DOI:** 10.3390/ijms22169056

**Published:** 2021-08-22

**Authors:** Ada Połońska, Katarzyna Jasieniecka-Gazarkiewicz, Lingjie You, Xiahui Hao, Sylwia Klińska, Yangmin Gong, Antoni Banaś

**Affiliations:** 1 Intercollegiate Faculty of Biotechnology, University of Gdansk and Medical University of Gdańsk, 80-307 Gdansk, Poland; katarzyna.jasieniecka@biotech.ug.edu.pl (K.J.-G.); sylwia.klinska@phdstud.ug.edu.pl (S.K.); 2 Oil Crops Research Institute, Chinese Academy of Agricultural Sciences, Wuhan 430062, China; you15826722881@126.com (L.Y.); haoxiahui221@126.com (X.H.); gongyangmin@caas.cn (Y.G.); 3 Key Laboratory of Biology and Genetic Improvement of Oil Crops, Ministry of Agriculture, Wuhan 430062, China

**Keywords:** LPCAT, VLC-PUFA, eicosapentaenoic acid, eicosatetraenoic acid, diatoms, *Phaeodactylum tricornutum*

## Abstract

The search of the *Phaeodactylum tricornutum* genome database revealed the existence of six genes potentially encoding lysophospholipid acyltransferases. One of these genes, Phatr3_J20460, after introduction to yeast *ale1* mutant disrupted in the LPCAT gene, produced a very active acyl-CoA:lysophosphatidylcholine (LPCAT) enzyme. Using in vitro assays applying different radioactive and non-radioactive substrates and microsomal fractions from such yeast, we have characterized the biochemical properties and substrate specificities of this *Pt*LPCAT1. We have found that the substrate specificity of this enzyme indicates that it can completely supply phosphatidylcholine (PC) with all fatty acids connected with a biosynthetic pathway of very long-chain polyunsaturated fatty acids (VLC-PUFAs) used further for the desaturation process. Additionally, we have shown that biochemical properties of the *Pt*LPCAT1 in comparison to plant LPCATs are in some cases similar (such as the dependency of its activity on pH value), differ moderately (such as in response to temperature changes), or express completely different properties (such as in reaction to calcium and magnesium ions or toward some acyl-CoA with 20C polyunsaturated fatty acids). Moreover, the obtained results suggest that cloned “Phatr3_J20460” gene can be useful in oilseeds plant engineering toward efficient production of VLC-PUFA as LPCAT it encodes can (contrary to plant LPCATs) introduce 20:4-CoA (n-3) to PC for further desaturation to 20:5 (EPA, eicosapentaenoic acid).

## 1. Introduction

Diatoms are an abundant group of eukaryotic photosynthetic microalgae, which belong to the pennates (*Bacillariophyceae*) class. They occur mainly in marine and freshwater environments. Among diatoms, *Phaeodactylum tricornutum* was the first with a completely sequenced genome [1]. *P. tricornutum* has the ability to produce significant amounts of omega-3 very long-chain polyunsaturated fatty acids (VLC-PUFAs), especially eicosapentaenoic acid (EPA; 20:5^Δ5,8,11,14,17^) constituting up to 30% of total fatty acids [2].

Omega-3 VLC-PUFAs such as EPA and docosahexaenoic acid (DHA; 22:6^∆4,7,10,13,16,19^) are essential dietary nutrients with numerous beneficial effects on human health. Consumption of these fatty acids helps in preventing the onset of cardiovascular diseases, age-associated declines in cognition and promotes visual system development [3]. The main source of EPA and DHA in the human diet is marine fish. Accelerating overexploitation of marine stocks and contamination of marine environments call for finding new sources of omega-3 VLC-PUFAs [4]. The marine diatoms (as the primary producers of omega-3 VLC-PUFAs) have become the main candidate for the investigation of genes encoding the enzymes (desaturases and elongases) taking part in the biosynthesis of these high-value polyunsaturated fatty acids [5].

The biosynthesis of EPA and DHA can occur via two different pathways called “conventional” Δ6-pathway and “alternative” Δ8-pathway. In *P. tricornutum* and other marine microorganisms, the “conventional” Δ6-pathway is the most active [4].

The 18:1 (oleic acid) desaturation was established as the starting point of VLC-PUFAs biosynthesis. Subsequent reactions rely on a series of alternating desaturation and elongation steps. Enzymes responsible for the production of VLC-PUFAs, fatty acids composed of more than 18 carbon atoms, are located in the endoplasmic reticulum [1]. The EPA biosynthetic pathway was reconstituted in yeast using genes coding Δ5-desaturase, Δ6-desaturase from *P. tricornutum,* and Δ6-elongase from *Physcomitrella* *patens*. Further investigations of the substrate selectivity of Δ5- and Δ6- desaturases revealed that they convert omega-3 and omega-6 substrates with similar efficiencies [6]. Using pulse-chased experiments with [^14^C] fatty acids, Arao and Yamada [7] proposed the predominant route of EPA biosynthesis in *P. tricornutum*. The first step in this pathway is Δ6-desaturation of linoleic acid (LA; 18:2^Δ9,12^) to γ-linolenic acid (GLA; 18:3^Δ6,9,12^), which is then Δ15-desaturated to produce stearidonic acid (SDA; 18:4^Δ6,9,12,15^). SDA is elongated by two carbons and desaturated to yield eicosapentaenoic acid (EPA; 20:5^Δ5,8,11,14,17^). In diatoms that can synthesize DHA, created EPA can undergo the second step of elongation to docosapentaenoic acid (DPA; 22:5^Δ7,10,13,16,19^) by the action of Δ5-elongase. Subsequently, DPA can be desaturated to DHA by Δ4-desaturase. However, *P. tricornutum* produces DHA in trace levels only [6]. After biosynthesis in the endoplasmic reticulum, EPA can be transported to the plastid and incorporated into galactolipids, especially MGDG and DGDG [8,9]. The mechanism of trafficking EPA between the endoplasmic reticulum and plastid is unclear and requires further investigations [1,9]. It is speculated that direct contact of the outer plastidial membrane and ER membrane can facilitate EPA translocation [1].

The family of enzymes called acyl-CoA:lysophospholipids acyltransferase (LPLAT) occurs commonly in animals, plants, and microorganisms. LPLATs can be divided into groups named, e.g., LPAAT, LPEAT, LPCAT. These groups of enzymes differ from each other in substrate specificity toward fatty acid acceptors. LPAAT preferentially uses LPA and LPEAT—LPE as fatty acid acceptors. LPCAT group of enzymes is characterized by their highest specificity for lysophosphatidylcholine [10,11]. LPLATs in forward reaction use lysophospholipids (LPLs) and acyl-CoA to produce phospholipids (PLs). In reverse reaction, they produce LPL and acyl-CoA from PL and coenzyme A. LPCATs play the most important role in supplying the cytosolic acyl-CoA pool with acyl groups from PL. They catalyze preferentially the turnover of acyls between PC (the main membrane lipids and place of fatty acids modification) and acyl-CoA pools [10,11,12,13]. The genes coding LPCAT enzymes were cloned, and LPCATs from various plants were characterized, e.g., *Arabidopsis thaliana* [14], *Brassica napus* [15], *Nicotiana benthamiana* [16], *Ricinus communis* [7], *Hiptage benghalensis*, *Lesquerella fendleri*, *Carthamus tinctorius* [12], and *Helianthus annuus* [17].

Synthesis of EPA in *P. tricornutum* is highly efficient, which implies that this diatom has developed a high ability to channel intermediates of its biochemical pathway between different lipid pools [8]. Desaturases from *P. tricornutum* are phospholipid-dependent, as opposed to elongases, which are acyl-CoA-dependent [18]. Therefore, it is predicted that this organism has a highly efficient mechanism of acyl exchange between PC pool and acyl-CoA pool catalyzed by the action of LPCATs, similar to the one present in plants [8].

Transgenic oilseed plants with introduced EPA and DHA biosynthetic pathways derived from marine diatoms or other microorganisms synthesizing such fatty acids could become alternative sources of omega-3 VLC-PUFA. Many attempts have been made to produce such transgenic plants. However, low product accumulation obtained so far necessitates further improvement to allow for profitable industrial-scale production of such fatty acids [19]. That low efficiency indicates some metabolic bottlenecks in these transgenic plants. One of these bottlenecks could be the substrate dichotomy between desaturases and elongases derived from diatoms. It is suggested that LPCAT type of enzymes from marine organisms might overcome substrate dichotomy by switching intermediates between acyl-CoA pool and PC. The action of these enzymes may lead to increased yields of EPA in transgenic plants due to potentially different characteristics of LPCATs from marine diatoms compared to those find in plants [20].

LPCATs from microalgae have not been characterized yet, so in this study, we investigate the biochemical properties, positional specificity, and substrate specificity of *Pt*LPCAT1 cloned by us. Additionally, we investigated substrate specificity of LPCATs of a microsomal fraction of developing seeds of *Camelina sativa* toward different acyl-CoA with polyunsaturated fatty acids. Based on the results of this study and literature data, we discuss the similarities and differences between LPCATs from diatoms and plants.

## 2. Results

### 2.1. Sequence analysis of Phaeodactylum tricornutum LPCAT

**Figure 1:**
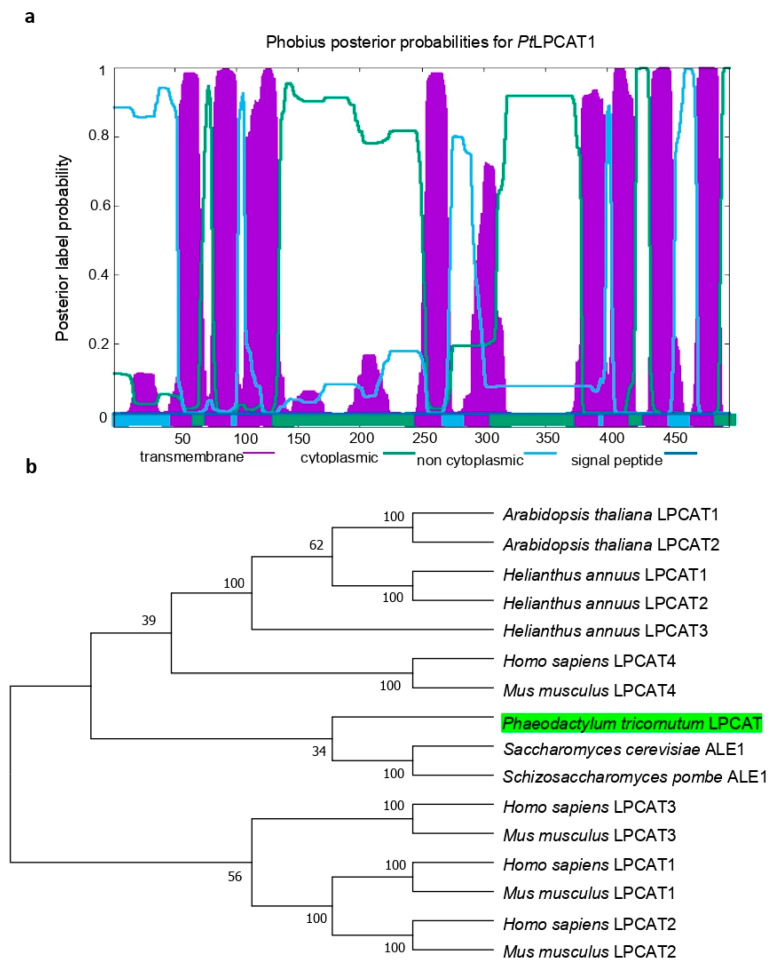
Sequence analysis of *Pt*LPCAT1. (**a**) Predicted transmembrane domain for *Pt*LPCAT1. The TMHMM web tool plots the probability of the sequence forming a transmembrane helix (0–1.0 on the *y*-axis). Regions of the *Pt*LPCAT1 sequence predicted to be transmembrane, cytoplasmic, and non-cytoplasmic are shown in purple, green, and light blue, respectively. (**b**) Phylogram of the LPCAT family showing 16 proteins from diatom, yeast, human, mouse, and plants. The candidate LPCAT of the diatom *Phaeodactylum tricornutum* (*Pt*LPCAT1) in this study is highlighted with a green box. Bootstrap values are indicated in %.

To identify the putative LPCAT proteins from the diatom *P. tricornutum*, we performed the search of the *P. tricornutum* genome database using the BLAST program with the yeast and Arabidopsis LPCAT acyltransferase sequences. As a result, six proteins were found to contain the lysophospholipid acyltransferase (LPLAT) domain or identified as acyltransferases of the membrane-bound O-acyltransferase (MBOAT) family. We focused on Phatr3_J20460 based on the experiment of complementation of the yeast mutant Y02431 (Table S1) and its closer phylogenetic relationship with the LPCAT family proteins. This candidate LPCAT was designated *Pt*LPCAT1, and it has a predicted molecular weight of 56.9 kDa and a theoretical isoelectric point (*pI*) of 8.55. Further prediction of transmembrane structure for *Pt*LPCAT1 revealed eight regions containing potential transmembrane sequences (Figure 1a), indicating it is a highly transmembrane acyltransferase. The search for the putative signal sequences using PredSL and PSORTII showed that *Pt*LPCAT1 has a predicted N-terminal signal peptide (cleavage site at 42) and localization in the endoplasmic reticulum (ER) and plasma membrane (44% for the *k*-NN prediction). These analyses indicate that *Pt*LPCAT1 might be localized in the ER membrane. Phylogenetic analysis was performed with amino acid sequences of putative LPCATs from yeast, diatom, mouse, human, and plants. The phylogenetic tree showed that the analyzed LPCATs are related to a broad range of sequences, i.e., *P. tricornutum Pt*LPCAT1 is most closely related to the yeast LPCATs from *S. cerevisiae* and *S. pombe*, followed by two human and mouse LPCAT4 proteins, which form clusters with plant LPCATs from *A. thaliana* and *H. annuus.* Six other LPCAT proteins (LPCAT1, LPCAT2, and LPCAT3), from mouse and human, created a more distantly related group (Figure 1b). The *Pt*LPCAT1 sequence was distantly related to plant and animal counterparts, suggesting a diatom-specific divergence of the LPCAT family.

### 2.2. In Vitro Activity of Tested P. tricornutum LPCAT Enzyme

**Figure 2:**
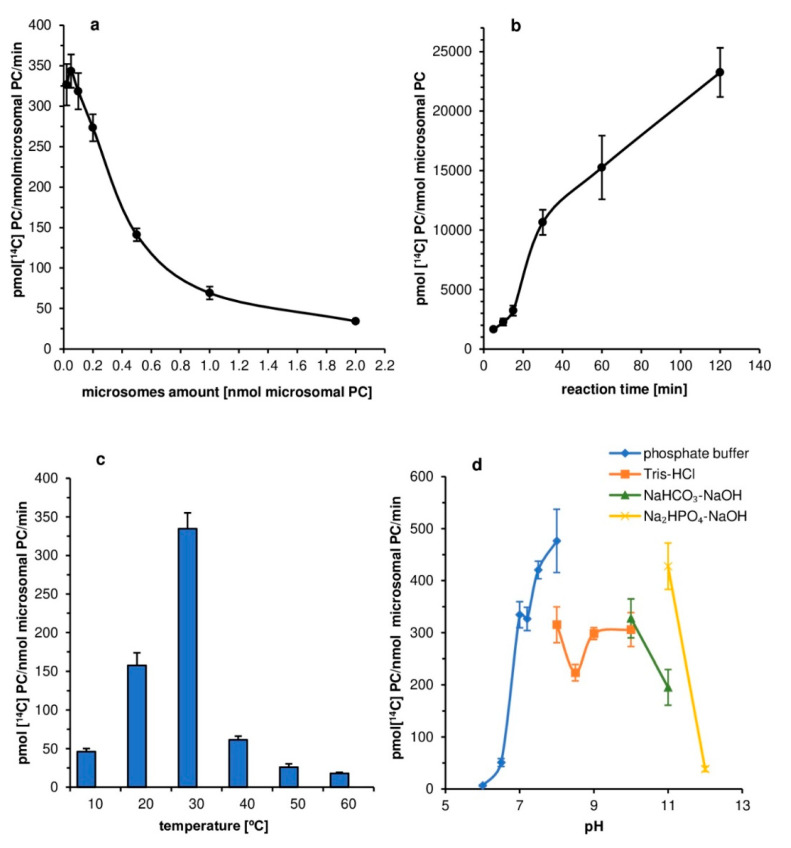
Effect of various factors on the activity of the tested acyl-CoA:lysophosphatidylcholine acyltransferase (*Pt*LPCAT1) of *Phaeodactylum tricornutum*. (**a**) Microsomal fraction “concentration” dependency. (**b**) Time dependency. (**c**) Temperature dependency. (**d**) pH dependency. Mean values and SD are presented (data from at least three independent assays).

Experiments started with assays establishing the optimal concentration of microsomal fraction for assaying *Pt*LPCAT1 activity. The maximum activity (calculated as: pmol [^14^C]PC/nmol microsomal PC/min) of the tested enzyme was observed when aliquots of microsomal fractions containing 0.05 nmol of microsomal PC were added to the assays. The activity dropped only slightly in the case of aliquots containing 0.02 and 0.1 nmol of microsomal PC. Further increases of microsomal membrane concentration to 0.5, 1, and 2 nmol of microsomal PC/assay caused a progressive reduction in the activity of the tested enzyme to 41%, 20%, and 10% (respectively) of its maximum level (Figure 2a).

In assays measuring the effect of reaction time on the activity of the tested enzyme, we observed three different periods of its activity; between: 0 and 15, 15 and 30, and 30 and 120 min (Figure 2a). The lowest activity was observed in the last period. However, the reaction runs at that time period nearly linearly. In the first period, the reaction was very fast in the first 5 min and then slowed down in the next 10 min. The highest activity occurred between 15 and 30 min of reaction time. The fluctuation of activity in the first 30 min of reaction was probably connected with membrane and substrate rearrangement in the assays. Nevertheless, at that time period, the overall activity was the highest; thus, we chose this time period for the subsequent optimization and substrate specificity assays.

The activity of *Pt*LPCAT1 was highly dependent on the reaction temperature. The highest activity was observed at 30 °C. Any decrease or increase in temperature generated a significant reduction in its activity. Assays carried out at 20 °C resulted in activity reduced to about 47% and at 40 °C to about 18%, of its maximum level. The activity in assays run at 10 °C reached only about 12%, and in assays carried out at 50 °C and 60 °C, only a few percentages of that at 30 °C (Figure 2c).

Four buffers were used in the evaluation of the effect of pH on *Pt*LPCAT1 enzyme activity. Each buffer had different pH ranges: 0.1 M phosphate buffer—pH 6.0–8.0, 0.1 M Tris-HCl buffer—pH 8.0–10.0, 0.1 M NaHCO_3_-NaOH buffer—pH 10.0–11.0 and 0.1 M Na_2_HPO_4_-NaOH buffer—pH 11.0–12.0. The activity of the tested *Pt*LPCAT1 was negligible at pH 6.0 and only a bit higher at pH 6.5. Increasing pH value to 7.0 had a strong acceleration effect on the enzyme activity; its activity increased about seven times compared to that at pH 6.5. The maximum level of activity of *Pt*LPCAT1 was reached at pH 8.0. Replacement of potassium phosphate buffer by Tris-HCl buffer decreased this activity by about 34% at mentioned pH value. The increase in pH to 8.5 caused a decline of its activity by about 30%. At pH 9.0, its activity returned to that from pH 8.0 and remained at that level also at pH 10.0. Replacement of Tris-HCl buffer by NaHCO_3_-NaOH buffer did not change activity at this pH; however, further increases of pH to 11.0 decreased the tested enzyme activity to about 41% of its maximum activity observed at pH 8.0 (with potassium phosphate buffer), activity roughly similar to that at pH 8.5. However, the use of Na_2_HPO_4_-NaOH buffer increased *P*tLPCAT1 activity at pH 11.0 to about 90% of its maximum activity. Higher alkalization of the environment (pH 12.0; Na_2_HPO_4_-NaOH buffer) resulted in the very low activity of the tested *Pt*LPCAT1 enzyme. Even though the *Pt*LPCAT enzyme had the highest activity at pH 8.0, for further analysis, we took pH 7.2 as the optimal one due to the fact that this pH is the most similar to that existing in diatom cells (Figure 2d).

**Figure 3:**
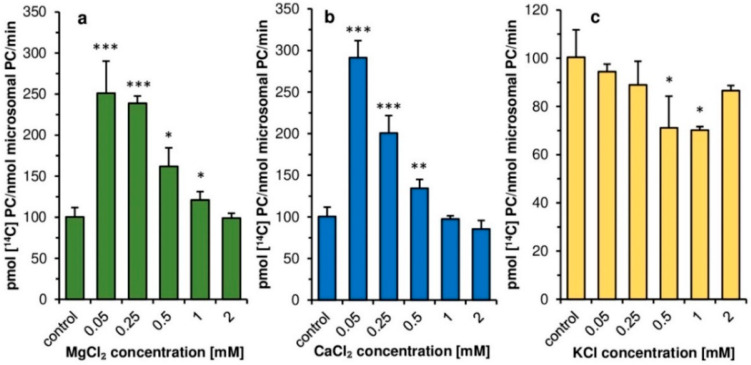
Effect of various ions on the activity of acyl-CoA:lysophosphatidiylcholine acyltransferase (LPCAT) of *Phaeodactylum tricornutum*. (**a**) Effect of magnesium ions. (**b**) Effect of calcium ions. (**c**) Effect of potassium ions. Mean values and SD are presented (data from at least three independent assays). Asterisk denotes significant differences between control (ions not added) and tested ions concentrations in a mean difference two-sided Student’s t-test: * *p* ≤ 0.05; ** *p* ≤ 0.01; *** *p* ≤ 0.001.

All three types of ions (Mg^2+^, Ca^2+^, and K^+^) affect the activity of *Pt*LPCAT1 (Figure 3). The presence of magnesium and calcium ions in the incubation buffer in 0.05 mM concentrations raised its activity by 151% and 191% (respectively) compared to assays without added ions. The increase in magnesium and calcium ions concentration to the range of 0.25–2.0 mM resulted in the weakening of their stimulatory effect. The increase in magnesium ions concentration to 0.25 mM reduced their effect by only 5%, but in the case of calcium by 31% as compared to 0.05 mM concentrations. Magnesium ions added to the assays at the highest tested concentration of 2.0 mM eliminated almost all stimulatory effects observed with lower concentrations. In the case of Ca^2+^ ions added to the assays in the concentration of 2 mM, the activity of the tested *Pt*LPCAT1 was even inhibited to 86% of activity obtained in control assays (Figure 3a,b). The addition of the potassium ions to the assays caused some inhibitory effect (at all tested concentration values) on the activity of the tested enzyme; the strongest inhibition was at a concentration of 0.5 mM (by 29%) (Figure 3c).

**Figure 4:**
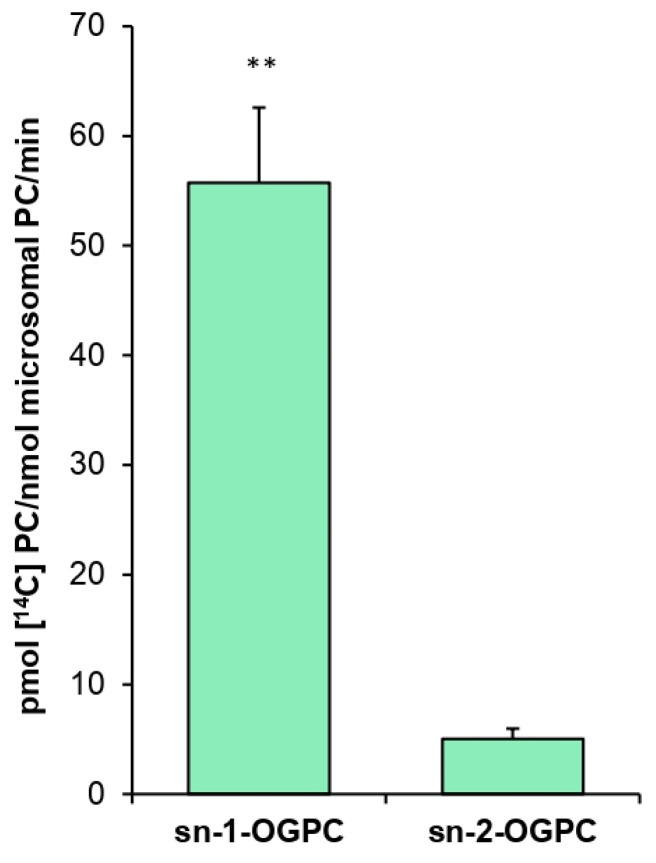
Activity of *P. tricornutum Pt*LPCAT1 toward the ether analog of *sn*-1-18:1-LPC (*sn*-1-18:1-O-GPC) and the ether analog of *sn*-2-18:1-LPC (*sn*-2-18:1-O-GPC) with [^14^C]18:1-CoA as acyl donor. Mean values and SD are presented (data from at least three independent assays). Asterisk denotes significant differences between activity toward sn-1-OGPC and sn-2-OGPC in a mean difference two-sided Student’s *t*-test: ** *p* ≤ 0.01.

The assays concerning positional specificity of *Pt*LPCAT1 were conducted with the ether analog of *sn*-1-18:1-LPC (*sn*-1-18:1-O-GPC) and the ether analog of *sn*-2-18:1-LPC (*sn*-2-18:1-O-GPC). Such an experiment showed that the tested enzyme can esterify fatty acids to both LPC positions, but its efficiency was 11 times higher toward the *sn*-2 position than toward the *sn*-1 position when 18:1-CoA was used as acyl donor (Figure 4).

### 2.3. Activity and Substrate Specificity of Tested P. tricornutum LPCAT Enzyme

**Figure 5:**
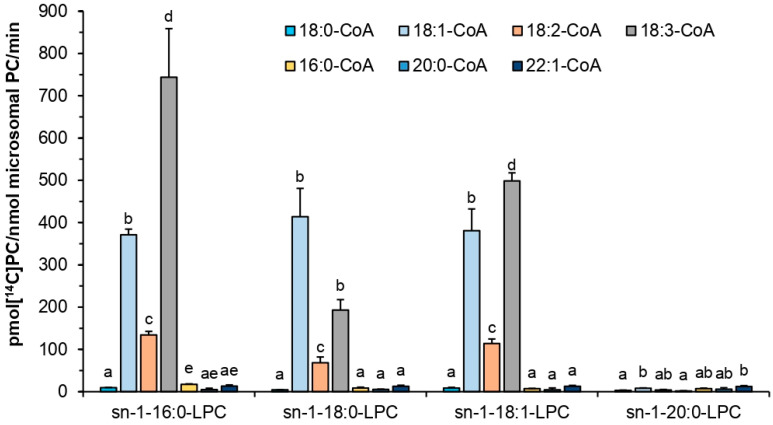
Activity of tested acyl-CoA:lysophosphatidylcholine acyltransferase (*Pt*LPCAT1) from *P. tricornutum* toward four different LPC in combination with seven different [^14^C]acyl-CoAs. Mean values and SD are presented (data from at least three independent assays). Statistical analysis of the difference in *Pt*LPCAT1 activity toward chosen acyl-CoA in assays with different acceptors was performed by one-way ANOVA followed by Tukey’s test. Different letters indicate a significant difference (*p* ≤ 0.05).

Subsequent assays measured *Pt*LPCAT1 activity toward four different LPC (*sn*-1-16:0-LPC, *sn*-1-18:0-LPC, *sn*-1-18:1-LPC, *sn*-1-20:0-LPC) in combination with seven different acyl-CoA ([^14^C]18:0-CoA, [^14^C]18:1-CoA, [^14^C]18:2-CoA, [^14^C]18:3-CoA, [^14^C]16:0-CoA, [^14^C]20:0-CoA and [^14^C]22:1-CoA). The highest activity was obtained when *sn*-1-16:0-LPC and [^14^C]18:3-CoA were added to the reaction mixture. The activities toward *sn*-1-18:0-LPC and *sn*-1-18:1-LPC in combination with [^14^C]18:3-CoA were significantly lower, accounting for about 26% and 67% of the activity with *sn*-1-16:0-LPC, respectively. *Pt*LPCAT1 activity was on quite a similar level, when 18:1-CoA was used as acyl donor and *sn*-1-16:0-LPC, *sn*-1-18:0-LPC, and *sn*-1-18:1-LPC as acyl acceptors, and was equal to 50%, 56%, and 51% (respectively) of the maximum activity reached when *sn*-1-16:0-LPC and [^14^C]18:3-CoA were used. The tested *Pt*LPCAT1 uses [^14^C]18:2-CoA in combinations with *sn*-1-16:0-LPC, *sn*-1-18:0-LPC, and *sn*-1-18:1-LPC much less efficiently compared to the two previously mentioned acyl donors; activity in these three types of assays was, respectively, about 18%, 9%, and 15% of activity reached when *sn*-1-16:0-LPC with [^14^C]18:3-CoA were used. The activity of the tested enzyme was very low in assays conducted with *sn*-1-20:0-LPC in combinations with all the tested acyl donors. Its efficiency was also marginal toward the [^14^C]saturated acyl-CoAs and [^14^C]22:1-CoA in combination with other tested LPC (*sn*-1-16:0-LPC, *sn*-1-18:0-LPC, *sn*-1-18:1-LPC) (Figure 5).

**Figure 6:**
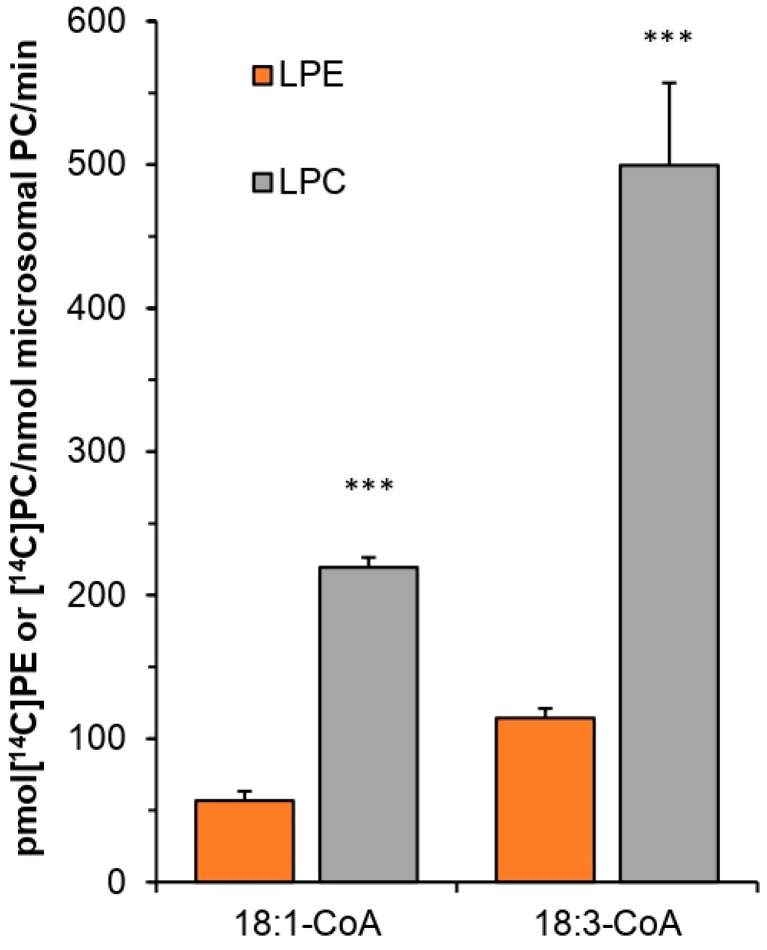
Activity of acyl-CoA:lysophosphatidylcholine acyltransferase (*Pt*LPCAT1) from *P. tricornutum* toward LPE and LPC in combination with [^14^C]18:1-CoA or [^14^C]18:3-CoA as acyl donor. Values shown are differences of activities of microsomal fractions of yeast Δ*ale1* expressing *Pt*LPCAT1 and activities of control yeast (Δ*ale1*) harboring an empty vector. Mean values and SD are presented (data from at least three independent assays). Asterisk denotes significant differences between activity toward LPE and LPC when [^14^C]18:1-CoA or [^14^C]18:3-CoA was used as acyl donor, in a mean difference two-sided Student’s *t*-test: *** *p* ≤ 0.001.

In assays studying *Pt*LPCAT1 activity toward: PA, LPE, LPC, LPG, and LPS, each of the above-mentioned lysophospholipid had 18:1 esterified to the *sn*-1 position. The results show that *Pt*LPCAT1 used the most efficiently LPC as acyl acceptor, both with [^14^C]18:1-CoA and [^14^C]18:3-CoA as acyl donors; however, the activity in assays with [^14^C]18:3-CoA was about 2.4 × higher compared to the activity with [^14^C]18:1-CoA. *Pt*LPCAT1 activity toward LPE accounted for approximately 24%–25% of its activity toward LPC with both used acyl donors. The activities toward LPA, LPG, and LPS were close to the detection threshold and are not presented in the figure (Figure 6).

**Figure 7:**
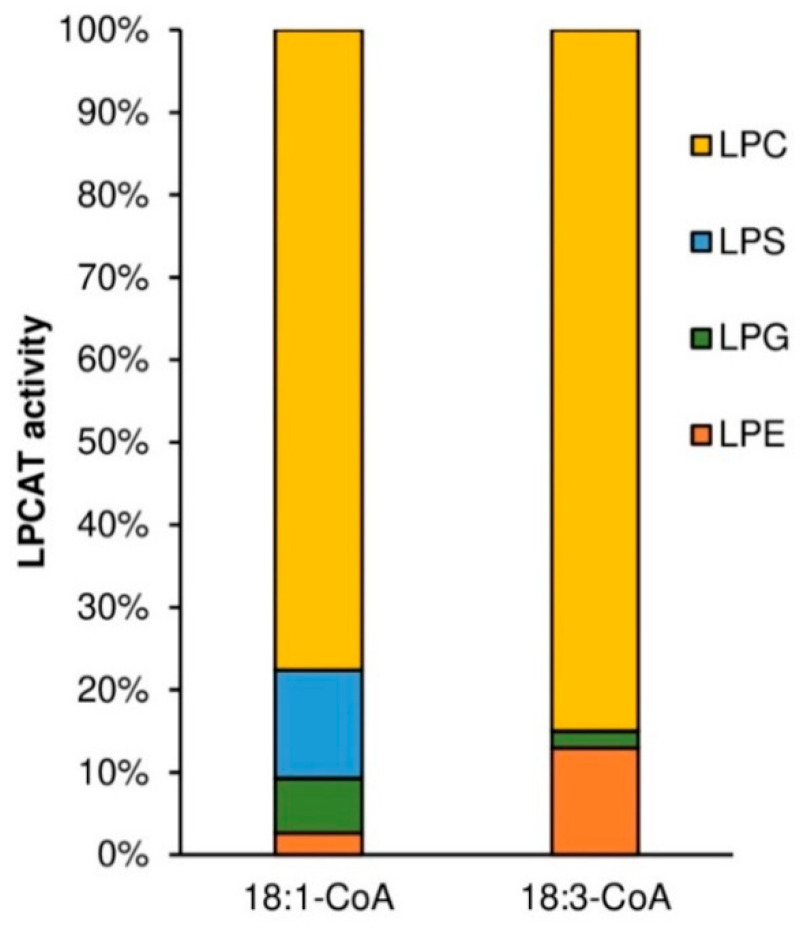
Activity of acyl-CoA:lysophosphatidylcholine acyltransferase (*Pt*LPCAT1) from *P. tricornutum* toward five different LPLs added to the reaction mixture together in equimolar concentration in combination with [^14^C]18:1-CoA or [^14^C]18:3-CoA as acyl donors. Values shown are differences of average activities of microsomal fractions of yeast Δ*ale1* expressing *Pt*LPCAT1 and average activities of control yeast (Δ*ale1*) harboring an empty vector (data from at least three independent assays).

Substrate selectivity assays with five LPLs (LPS, LPC, LPG, LPE, LPA) added to the reaction mixture together in equimolar concentration (1 nmol of each) in combination with [^14^C]18:1-CoA or [^14^C]18:3-CoA, showed a somewhat similar activity/specificity of the tested *Pt*LPCAT1 as in the assays with LPLs added to the reaction mixture separately. From the added LPLs, the tested enzyme preferentially used LPC independently of whether [^14^C]18:1-CoA or [^14^C]18:3-CoA was in the reaction mixture. Among the de novo synthesized phospholipids, [^14^C]PC accounted for approximately 73% in assays with [^14^C]18:1-CoA and for about 85% in assays with [^14^C]18:3-CoA. The use of the remaining lysophospholipids by the tested enzyme depended somewhat on the acyl-CoA used in the assay. In the case of [^14^C]18:3-CoA, only [^14^C]PE and [^14^C]PG were synthesized; about 13% and 2% of de novo synthesized [^14^C]PL. In assays with [^14^C]18:1-CoA, [^14^C]PS, [^14^C]PE, and [^14^C]PG appeared and accounted for approximately 13%, 9%, and 5%, respectively (Figure 7). Both, in assays with [^14^C]18:3-CoA and with [^14^C]18:1-CoA, the amount of de novo synthesized [^14^C]PA was at the same level as in corresponding assays with control microsomal fractions (prepared from Δ*ale1* yeast harboring an empty vector).

**Figure 8:**
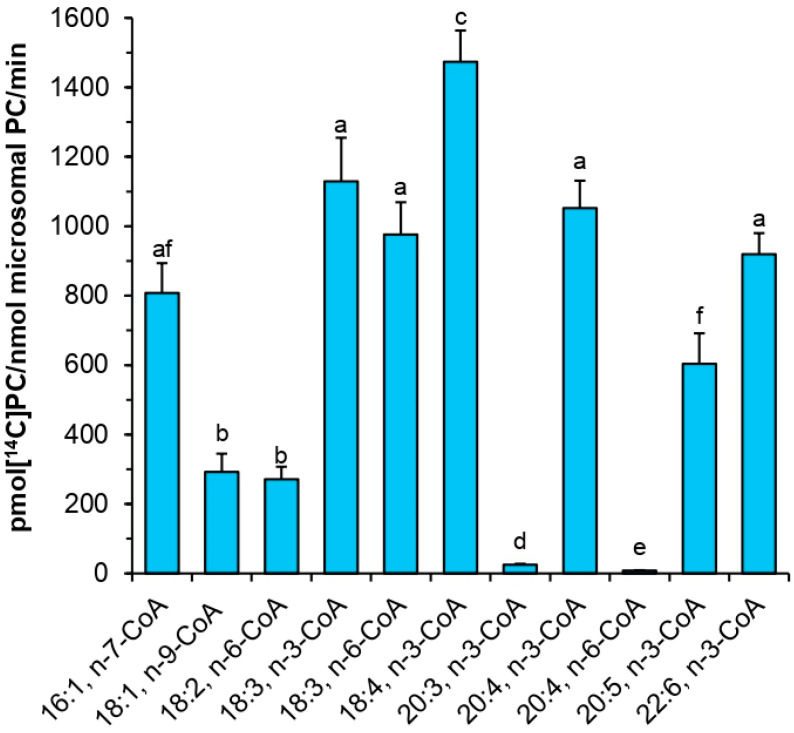
Activity of acyl-CoA:lysophosphatidylcholine acyltransferase (*Pt*LPCAT1) from *P. tricornutum* toward *sn*-1-[^14^C]18:1-LPC in combination with eleven different acyl-CoAs. Mean values and SD are presented (data from at least three independent assays). Statistical analysis was performed by one-way ANOVA followed by Tukey’s test. Different letters indicate a significant difference (*p* ≤ 0.05).

Detailed substrate specificity assays revealed high activity of *Pt*LPCAT1, especially toward omega-3 unsaturated fatty acids, which are intermediates of the DHA biosynthesis pathway. The best accepted acyl donor by *Pt*LPCAT1 was 18:4-CoA n-3 followed by: 18:3-CoA n-3, 20:4-CoA n-3, 18:3-CoA n-6, 22:6-CoA n-3, 16:1-CoA n-7, 20:5-CoA n-3, 18:1-CoA n-9, 18:2-CoA n-6 (listed in order of decreasing activity). *Pt*LPCAT1 activity toward 18:3-CoA n-3, 20:4-CoA n-3, 18:3-CoA n-6, 22:6-CoA n-3 and 16:1-CoA n-7 was ranging between 55% and 77%, of the activity toward 18:4-CoA n-3. The range of activity toward 20:5-CoA n-3, 18:1-CoA n-9, 18:2-CoA n-6 was between 18% and 41% of the activity toward 18:4-CoA n-3. On the contrary *Pt*LPCAT1 accepted 20:3-CoA n-3, 20:4-CoA n-6 and all tested saturated acyl-CoAs (16:0-CoA, 18:0-CoA, 20:0-CoA, 22:0-CoA, 24:0-CoA, 26:0-CoA, 14:0-CoA; data not shown) at very low or even marginal levels (Figure 8 and data not shown).

**Figure 9:**
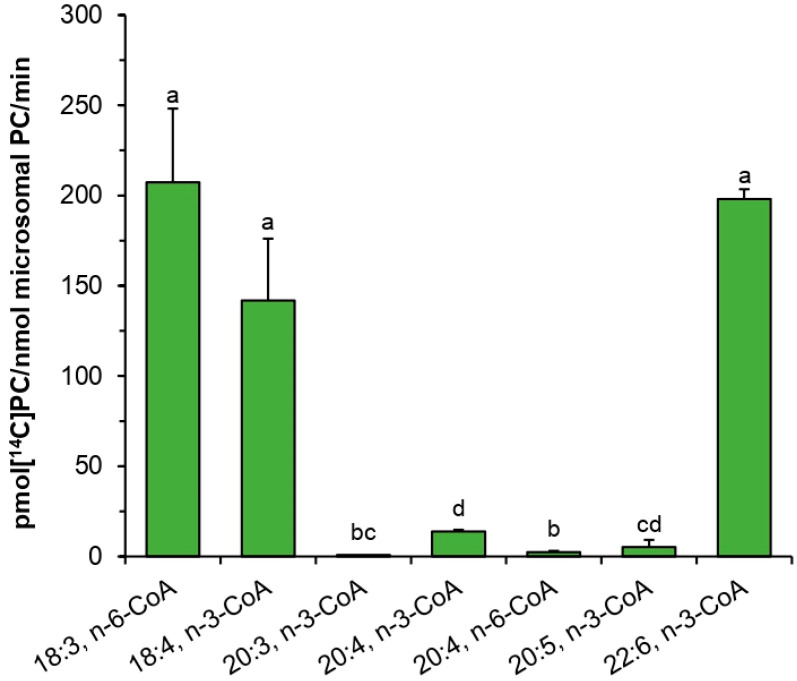
Activity of acyl-CoA:lysophosphatidylcholine acyltransferases (*Pt*LPCAT1) from a microsomal fraction of *C. sativa* developing seeds toward *sn*-1-[^14^C]18:1-LPC in combination with different unsaturated acyl-CoAs. Mean values and SD are presented (data from at least three independent assays). Statistical analysis was performed by one-way ANOVA followed by Tukey’s test. Different letters indicate a significant difference (*p* ≤ 0.05).

In assays with a microsomal fraction of developing seeds of *C. sativa, sn*-1-[^14^C]18:1-LPC in combination with several acyl-CoA with polyunsaturated fatty acids were used as substrates of LPCAT type of enzymes. From the tested substrate combinations, 18:3-CoA n-6, 22:6-CoA n-3, and 18:4-CoA n-3 were used with efficiency similar to 18:1-CoA n-9, 18:2-CoA n-6, and 18:3-CoA n-3 in assays performed by Klińska et al. [11] with the same kind of microsomal fractions. However, all tested acyl-CoA with 20C polyunsaturated fatty acid were very badly accepted by these LPCATs (Figure 9).

## 3. Discussion

Until now, biochemical characterization of LPLAT enzymes from diatoms has not been performed. Nevertheless, putative genes encoding LPLAT enzymes have been identified in the *P. tricornutum* genome by homology in a genome database [21]. In our studies, we have cloned six such genes, which, after introduction to yeast *ale1* mutant, passed the lyso-PAF sensitivity test. From these six genes, only “Phatr3_J20460” produced in the yeast system a very active enzyme with LPCAT activity. In the presented work, we have characterized the biochemical properties and substrate specificities of this *Pt*LPCAT1 using in vitro assays applying different radioactive and non-radioactive substrates and microsomal fractions from yeast *ale1* mutant harboring the “Phatr3_J20460” gene as an enzyme source.

In contrast to diatom, the plant LPCAT enzymes have been much better characterized [11,12,17]. Currently, it is considered that they are, at least partially, responsible for controlling of acyl composition of membrane phospholipids (particularly phosphatidylcholine) and cytosolic acyl-CoA pool via forward and backward reactions. It is assumed that they are one of the most important players in supplying cytosolic acyl-CoA pool with polyunsaturated fatty acids used further in phospholipids acyl editing process, storage lipid biosynthesis, or for elongation [11,12]. Mühlroth et al. [22] speculated that in diatoms, LPCAT type of enzymes could also play an important role in polyunsaturated fatty acids flux between PC and cytosolic acyl-CoA pool during the very long-chain polyunsaturated fatty acid biosynthesis process. They assumed, however, that most likely, these enzymes have different substrate specificities in comparison to the plant LPCATs.

In the presented studies, we have shown that *Pt*LPCAT1 was active toward various acyl-CoA with unsaturated fatty acids. The highest activity was observed toward 18:4-CoA n-3, and still high toward 18:3-CoA n-3 > 20:4-CoA n-3 > 18:3-CoA n-6 > 22:6-CoA n-3 > 16:1-CoA n-7 > 20:5-CoA n-3 > 18:1-CoA n-9 > 18:2-CoA n-6. Contrary to that, acyl-CoA with saturated fatty acids was almost not accepted by the enzyme. Surprisingly, its activity toward 20:3-CoA n-3 and toward 20:4-CoA n-6 was also very low. Thus, it seems that for the tested *Pt*LPCAT1 activity, not only the presence of double bonds is important, but also their place in the acyl moieties. When the first double bond is located further than at Δ9 position, such as in the mentioned 20:3-CoA (first double bond is at Δ11 position) or the last double bond is located closer than at n-3 in fatty acids longer than 18C (such as in 20:4-CoA n-6) it seems that acyl-CoA with such fatty acids cannot be accepted (or are used very badly) by the enzyme. The tested *Pt*LPCAT1 activity toward acyl-CoA with unsaturated C18 acyl groups was similar to the substrate specificity of plant LPCATs [11,12]. However, some differences were noted in the case of saturated fatty acids. Plant LPCATs seem to be able to better use such acyl-CoA. It was shown that *B. napus* and *C. sativa* LPCATs exhibited activity toward 16:0-CoA and 18:0-CoA [11,16] higher than the tested *Pt*LPCAT1. The use of acyl-CoA with VLC-PUFA by plant LPCATs was not tested so far. Klińska et al. [11] showed that *C. sativa* LPCATs cannot use 20:1-CoA and 22:1-CoA; however, fatty acids in these acyl-CoA have the first double bond in a position further than at Δ9; thus, the situation can be similar such as with 20:3-CoA (n-3) tested in this study. The data obtained in the presented studies revealed, however, that at least LPCATs of microsomal fractions of *C. sativa* developing seeds cannot use not only 20:3-CoA (n-3) and 20:4-CoA (n-6) such as *Pt*LPCAT1 but also 20:4-CoA (n-3) and 20:5-CoA (n-3).

Obtained results are confirmation of earlier assumptions that *Pt*LPCATs may play an important role during the VLC-PUFAs biosynthetic pathway. Tested *Pt*LPCAT1 showed high substrate specificity toward acyl-CoA derivatives of 20:4 (ETA, n-3). This is in agreement with the proposed biosynthesis pathway of EPA and DHA in *P. tricornutum* through 20:4 (n-3), but not by 20:4 (n-6) [1]. Taking into consideration that the Δ5 desaturation of 20:4 (ETA) to 20:5 (EPA) takes place in PC, and it is the last step of the EPA biosynthesis pathway, the preceding action of *Pt*LPCAT1 can play the major role in the production of the high level of EPA in *P. tricornutum*. Moreover, ETA fatty acid constitutes only approximately 2.2% of the fatty acid profile of *P. tricornutum* cultivated under normal growth conditions [1]; therefore, ETA fatty acids are probably quickly introduced to PC for desaturation by the action of *Pt*LPCAT1. These findings could suggest that the tested *Pt*LPCAT1 can potentially eliminate the acyltransferase bottlenecks in transgenic oilseed plants producing EPA and DHA fatty acids [23], as at least LPCATs of *C. sativa* seeds cannot use 20:4-CoA (n-3), which is the key step in the biosynthetic pathway of EPA and in consequence also in DHA fatty acids.

In the biosynthesis of VLC-PUFAs, not only the introduction of proper acyl-CoA to the PC pool for the desaturation process is important, but also the transfer of some intermediates after modification in PC to cytosol for elongation. The tested *Pt*LPCAT1 can also be involved in this process. It has been proven that these types of enzymes in the backward reaction can transfer fatty acids from PC to the acyl-CoA pool (see Introduction). We have not tested this, however, in this study. It will be one of the subjects of our future research. It will require some modifications of microsomal membranes used in the studies with PC species containing different very long-chain polyunsaturated fatty acids. We are currently developing the precise protocol for performing these experiments.

The positional specificity of *Pt*LPCAT1 proved to be 11 times higher toward the *sn*-2 position than toward the *sn*-1 position of LPC when 18:1-CoA was used as a fatty acid donor. A similar result was obtained, for example, in an experiment conducted by Lager et al. [12], who demonstrated that LPCAT1 and LPCAT2 from *A. thaliana* are 7–8 times more efficient toward the *sn*-2 position than toward the *sn*-1 position of LPC with 18:1-CoA as acyl donor. Klińska et al. [11] arrived at the same conclusion in the case of *C. sativa,* which LPCATs were approximately eight times more active toward *the sn*-2 position. These data indicate that the positional specificity of the tested *Pt*LPCAT1 does not differ significantly from that of plant LPCATs.

Similar to plant and yeast LPCATs, the tested *Pt*LPCAT1 also showed specificity toward different lysophospholipids [10,14]. Similar to the previously mentioned LPCATs, the highest activity was toward LPC. However, the differences in specificity toward, e.g., LPC and LPE were smaller than presented by plant LPCATs and much more similar to specificity presented by yeast ALE1 in single LPL assays (the competence tests with a mixture of different LPLs were not performed for other LPCATs). From among different LPC used in our assays, the tested *Pt*LPCAT1 preferentially used *sn*-1-16:0-LPC and with somewhat lower intensity *sn*-1-18:1-LPC and *sn*-1-18:0-LPC and was almost not active toward *sn*-1-20:0-LPC. The previous research concerning plant LPCATs was almost exclusively based on *sn*-1-18:1-LPC [10,11,14]. Lager et al. [12] usage of *sn*-1-ric-LPC (LPC with ricinoleic acid) resulted in a lower affinity of tested plant LPCATs than toward *sn*-1-18:1-LPC.

The tested *Pt*LPCAT1 was the most active at 30 °C, and its activity sharply decreased at either higher or lower temperatures. The 30 °C was also the optimal temperature for *C. sativa* LPCATs; however, in that case, the temperatures below and higher than optimal caused less drastic changes [11]. The physiological significance of this high-temperature dependency of *Pt*LPCAT1 is unknown. However, for *P. tricornutum* the temperatures below 15 °C and above 21 °C are regarded as stress conditions [24]. Yongmanitchai and Ward [25] reported that the EPA production is most effective in *P. tricornutum* in a temperature range of 21.5–23 °C; however, the optimal temperature for growth is estimated as 15 to 20 °C. The observed doubling of activity of *Pt*LPCAT1 between 20 and 30 °C could be in some way connected with this stress increase in EPA production.

The pH-level dependency of the analyzed diatom LPCAT activity was somewhat similar to that expressed by LPCATs of *C. sativa* seeds’ microsomal fraction [11]. Both types of LPCAT were most active in alkaline pH (pH 8–11); however, some differences in the dependency curves were noted. The maximum LPCAT activity in alkaline pH was also reported in the case of *B. napus* [26,27] and mice [28]. Thus, it seems to be a common feature of LPCAT type of enzymes, with, however, yet unknown physiological significance. When *P. tricornutum* is cultivated in high alkaline pH, cells are mobilized to produce more lipids than during the cultivation in neutral pH [29,30]. However, so far, there is no evidence to connect this with the high activity of LPCAT at such pH.

Contrary to plant LPCATs [11], the addition of magnesium and calcium ions at concentrations 0.05–0.5 mM had a clear stimulatory effect on the activity of *Pt*LPCAT1. Such calcium and magnesium concentrations, however, significantly inhibit LPCATs activity of developing seeds of *C. sativa*. Thus, in this aspect, plant and diatom LPCATs behaving completely differently. Similar to plant LPCATs, the potassium ions had some inhibitory effect on *Pt*LPCAT1 activity. However, the inhibitory effect was rather small in both cases, and depending on potassium ions, concentration ranged from a few to max 30% compared to assays without these ions. The higher stimulation of *Pt*LPCAT1 activity observed in lower magnesium or calcium ions concentrations compared to that observed at higher concentrations (≥0.5 mM) could be connected with the impeding of acyl-CoA solubility by high concentrations of these ions [31]

As the tested ions concentrations in the diatom cells (or different compartments of the cells) could be different from these used in our assays, we could not directly transfer the results obtained in vitro to the situation in vivo and rather treat these findings as features of the characterized enzyme. In the case of calcium ions, we expected that intracellular concentration could be close to the lowest concentrations used in our assays. In diatoms, calcium ions are actively transported from the cell by Ca^2+^-ATPase located in the cell membrane to maintain low intracellular concentration as opposed to their extracellular concentration [32]. In the case of magnesium and potassium ions, their intracellular concentration might be higher than used in our assays. Dickson and Kirst [33] showed, for instance, that the intracellular concentration of K^+^ and Mg^2+^ in *P. tricornutum* was about 200 and 32 mM, respectively, when cells were cultured in media with salinity corresponding to that existing in the natural habitat. Similarly, a high concentration of K^+^, accounting for about 140 mM were observed by Overnell [34] in the cells of *P. tricornutum* grew in medium with potassium concentration at 6.7 mM. The concentrations of tested ions could be, however, different in direct environment of the enzyme and could be more similar to that used in our assays. Nevertheless, we should have in mind that calcium, magnesium and potassium ions could affect *Pt*LPCAT1 activity.

To sum up, we can state that the substrate specificity of the tested *Pt*LPCAT1 indicated that it can completely supply PC with all fatty acids connected with DHA biosynthetic pathway for the desaturation process. However, the biosynthetic process of VLC-PUFA in diatoms also requires the transfer of fatty acids from PC to cytosolic acyl-CoA pool for the elongation process. The involvement of the tested *Pt*LPCAT1 in this process needs to be elucidated in further studies. We can also mention that biochemical properties of the tested *Pt*LPCAT1 are in some cases (such as the dependency of its activity on pH value) similar, differ moderately (such as in response to temperature changes), or express completely different properties (such as in reaction to calcium and magnesium ions or toward some acyl-CoA with 20C polyunsaturated fatty acids) compared to plant LPCATs.

## 4. Materials and Methods

### 4.1. Chemicals

[^14^C]-labeled fatty acids were purchased from PerkinElmer Life Science (Waltham, MA, USA). *sn*-1-18:1-*sn*-2-[^14^C]18:2-phosphatidic acid, *sn*-1-18:1-*sn*-2-[^14^C]18:2-phosphatidylethanolamine and *sn-*1-18:1-*sn*-2-[^14^C]18:2-phosphatidylcholine used as TLC standard were synthesized biochemically with use of a microsomal fraction of yeast overexpressing ALE1 and *sn*-1-18:1-LPA, *sn*-1-18:1-LPE or *sn*-1-18:1-LPC together with [^14^C]18:2-CoA as substrates in assays system described below. Synthesized [^14^C]-phospholipids were separated on TLC and eluted to chloroform as described below for separation of [^14^C]-LPC. *sn*-1-[1-^14^C]18:1-LPC was prepared by phospholipase A_2_ (Sigma-Aldrich, St. Louis, MO, USA) treatment of *sn*-1-*sn*-2-[1-^14^C]18:1-phosphatidylcholine purchased from American Radiolabeled Chemicals (Saint Louis, MO, USA). Reaction products were separated on TLC plates in polar solvent system consisting of chloroform:methanol:acetic acid:water (90:17,5:10:3,5; *v*:*v*:*v*:*v*). The radioactive LPC was eluted from silica gel with methanol:chloroform (2:1; *v*:*v*) by sonication and was subsequently extracted according to the method of Bligh and Dyer [35]. Non-radioactive fatty acids and lysophospholipids were obtained from Larodan Fine Chemicals (Malmö, Sweden). The [^14^C]acyl-CoAs and non-radioactive acyl-CoAs were synthesized by the method described by Sánchez et al. [36] with modifications. Other chemicals were obtained from Sigma-Aldrich or Merck (Darmstadt, Germany).

### 4.2. Gene Cloning and Sequence Analysis

The coding sequence of the LPCAT candidate gene (*Pt*LPCAT1, Phatr3_J20460) was retrieved from the *P. tricornutum* genome database (http://protists.ensembl.org/Phaeodactylum_tricornutum/Info/Index/; accessed on 11 July 2018), and amplified by reverse-transcription PCR (RT-PCR) using total RNA as a template according to the supplier’s recommendations with gene-specific sense primer (5′-AAGCTTATGAGTCTCCCCGAAGCC-3′) and antisense primer (5′ TCTAGATTCTTTCTTTTCTTTCTTGGGCG-3′), which were equipped with terminal restriction sites of *Hind*III and *Xba*I, respectively. The ~1.5 kb DNA fragment produced by RT-PCR was ligated into the yeast expression vector pYES2/CT (Invitrogen; Waltham, MA, USA), sequenced, and introduced for heterologous expression into the *Saccharomyces cerevisiae* mutant Y02431 (Δ*lca/*Δ*ale1*) disrupted in endogenous LPCAT enzyme activity. To search for the orthologs from other organisms for the construction of a phylogenetic tree, we performed Basic Local Alignment Search Tool (BLAST) analysis using the amino acid sequence of *Pt*LPCAT1 as a query. Analysis of the presence of conserved acyltransferase domains was conducted using hmmer searches [37]. Prediction of the potential transmembrane domains within the primary sequence of *Pt*LPCAT1 protein was performed using Phobius (https://phobius.sbc.su.se/; accessed on 18 August 2021) [38]. The protein sequence was also analyzed with PredSL (http://aias.biol.uoa.gr/PredSL/input.html; accessed on 10 July 2021) and PSORTII (http://psort.hgc.jp/form2.html; accessed on 18 August 2021) to search for the presence of putative N-terminal targeting sequences. The theoretical molecular weight and isoelectric point (*pI*) of *Pt*LPCAT1 were computed by the Compute pI/Mw server (http://web.expasy.org/compute_pi/; accessed on 14 July 2021). For the construction of a phylogenetic tree of LPCATs, a total of 16 amino acid sequences were aligned using ClustalW [39] in the Alignment Explorer, MEGA 4.0 [40]. Gaps and ambiguously aligned regions were omitted from further analysis. Phylogenetic and molecular evolutionary analyses were performed using MEGA 4.0 version by the neighbor-joining method [41]. The bootstrap value of 1000 replicas was selected together with the JTT model. Amino acid sequences selected from GenBank were: *Phaeodactylum tricornutum* LPCAT, Phatr3_J20460 (The ID number for the diatom LPCAT is according to the *P. tricornutum* genome database); *Arabidopsis thaliana* LPCAT1, NP_172724; *Arabidopsis thaliana* LPCAT2, NP_176493; *Homo sapiens* LPCAT1, NP_079106; *Homo sapiens* LPCAT2, NP_060309; *Homo sapiens* LPCAT3, NP_005759; *Homo sapiens* LPCAT4, NP_620154; *Mus musculus* LPCAT1, BAE94687; *Mus musculus* LPCAT2, BAF47695; *Mus musculus* LPCAT3, BAG12120; *Mus musculus* LPCAT4, BAG12122; *Saccharomyces cerevisiae* ALE1, NP_014818; *Schizosaccharomyces pombe* ALE1, NP_596779; *Helianthus annuus* LPCAT1, AER57988; *Helianthus annuus* LPCAT2, AER57989; and *Helianthus annuus* LPCAT3, ARQ87991.

### 4.3. Microsomal Preparation and Enzymes Assays

The yeast Δ*ale1* cells, with introduced plasmids pYES2/CT or plasmids pYES2/CT harboring *Pt*LPCAT1 gene (Phatr3_J20460), were cultured with uracil dropout medium for 24 h. After that time, the yeast cultures were rejuvenated to an OD_600_ = 0.2 and grew for another 24 h. On the third day, galactose was added to the cultures in an amount equivalent to 2% of the final concentration. The yeast cells were grown for at least 24 h to an OD_600_ = 3–4 and harvested by centrifugation at 1500× *g* for 10 min. The pellets were resuspended in 20 mL of Tris buffer (25 mM Tris-HCl pH 7.9) in order to wash cells from the residue of the medium. Then yeast cells were collected once again and resuspended in 1.5 mL of glass bead disruption buffer (20 mM Tris-HCl, pH 7.9, 10 mM MgCl_2_, 1 mM EDTA, 5% glycerol, 0.3 M ammonium sulfate) with protease inhibitors (Complete Tablets, Roche). Glass disruption beads were added to the resuspended yeast cells, which were subsequently homogenized in Mini Bead Beater (BioSpec Products, Bartlesville, OK, USA). The homogenization process was carried out for 5 × 30 s, with 30 s breaks in between. After a 5 min break, the above process was repeated once again. The glass disruption beads and not crashed cells were separated from the homogenates by centrifugation for 10 min at 1500× *g*. Supernatants were filtered through Miracloth to ultra-centrifuge tubes and centrifugated at 100,000× *g* for 2 h. The pellets were washed with 0.1 M potassium phosphate buffer pH 7.2 (without vortexing) and homogenized with the addition of a new, small portion of potassium phosphate buffer in glass homogenizers. Obtained homogenates of microsomes were divided on aliquots and stored at −80 °C. To estimate microsomes “concentrations”, the content of microsomal endogenous phosphatidylcholine (PC) was evaluated with the use of TLC and gas chromatography analysis [11].

In addition to the yeast microsomal fractions, the microsomal fraction of *C. sativa* developing seeds was prepared according to the method described by Klińska et al. [11].

First assays aimed at optimization of the conditions of the in vitro reaction catalyzed by tested *Pt*LPCAT1 enzyme. The influence of factors such as the amount of microsomal fraction, reaction time, temperature, buffer pH, and effect of ions (K^+^, Ca^2+^, Mg^2+^) on the *Pt*LPCAT1 activity were investigated, as enzyme substrates 5 nmol of [^14^C]18:1-CoA (acyl donor) and 5 nmol of *sn*-1-18:1-lysophosphatidylcholine (acyl acceptor) were used in these assays. The reactions were carried out in Eppendorf tubes with 0.1 M potassium phosphate buffer (100 µL), with two exceptions. The first concerned assays aimed at estimation of the effects of pH on enzyme activity (used buffers are presented in the legend of Figure 2d). The second concerned assays measured the effect of selected ions on enzyme activity, where HEPES buffer (pH 7.2) was used, due to the danger of forming insoluble salts of Mg^2+^ and Ca^2+^ ions in potassium phosphate buffer.

Further studies concerned the specific activity of *Pt*LPCAT1 toward various substrates. Positional specificity was tested in assays containing 5 nmol of [^14^C]18:1-CoA and 5 nmol of one of the ether analogs: *sn*-1-18:1-lyso-PC (*sn*-1-O-GPC) or *sn*-2-18:1-lyso-PC (*sn*-2-O-GPC).

Substrate specificity of tested *Pt*LPCAT1 enzyme toward LPA, LPE, and LPC, was studied with two acyl donors [^14^C]18:1-CoA and [^14^C]18:3-CoA. Substrate selectivity assays were performed with LPA, LPE, LPC, and LPG added to the reaction in equimolar concentration, together with acyl donors: [^14^C]18:1-CoA or [^14^C]18:3-CoA. Activity toward four different LPC: *sn*-1-16:0-LPC, *sn*-1-18:0-LPC, *sn*-1-18:1-LPC, *sn*-1-20:0-LPC was measured with seven different [^14^C]acyl-CoA: palmitoyl-CoA ([^14^C]16:0-CoA), stearoyl-CoA ([^14^C]18:0-CoA), oleoyl-CoA ([^14^C]18:1-CoA), linoleoyl-CoA ([^14^C]18:2-CoA), linolenoyl-CoA ([^14^C]18:3-CoA), arachidoyl-CoA ([^14^C]20:0-CoA) and erucoyl-CoA ([^14^C]22:1-CoA). In these assays, 5 nmol of respective lysophospholipid and 5 nmol of respective acyl-CoA were added together to the reaction mixtures, with the exception of assays measuring substrate selectivity to which 1 nmol of each lysophospholipid was added.

Assays measuring the substrate specificity toward acyl donors were performed with twenty one non-radioactive acyl-CoAs: myristoyl-CoA (14:0-CoA), palmitoyl-CoA (16:0-CoA), palmitoleoyl-CoA (16:1^Δ9^-CoA), stearoyl-CoA (18:0-CoA), oleoyl-CoA (18:1^Δ9^-CoA), linoleoyl-CoA (18:2^Δ9,12^-CoA), α-linolenoyl-CoA (18:3^Δ9,12,15^-CoA), γ-linolenoyl-CoA (18:3^Δ6,9,12^-CoA), eicosatetraenoyl-CoA (18:4^Δ6,9,12,15^-CoA), arachidoyl-CoA (20:0-CoA), eicosenoyl-CoA (20:1^Δ11^-CoA), eicosatrienoyl-CoA (20:3^Δ11,14,17^-CoA), arachidonoyl-CoA (20:4^Δ5,8,11,14^-CoA), eicosatetraenoyl-CoA (20:4^Δ8,11,14,17^-CoA), eicosapentaenoyl-CoA (20:5^Δ5,8,11,14,17^-CoA), docosanoyl-CoA (22:0-CoA), erucoyl-CoA (22:1^Δ9^-CoA), docosapentaenoyl-CoA (22:6^Δ4,7,10,13,16,19^-CoA), tetracosanoyl-CoA (24:0-CoA), nervonoyl-CoA (24:1^Δ9^-CoA), and hexacosanoyl-CoA (26:0-CoA). The single reaction contained 5 nmol of respective acyl-CoA and 5 nmol of *sn*-1-[^14^C]18:1-LPC. The assays were carried out with 0.1 M potassium phosphate buffer (pH 7.2; 100 µL). Microsomal fractions (source of the tested enzyme) were added in amount equivalent to 0.05 nmol of microsomal PC (approximately 0.22 µg of microsomal proteins). Assays were incubated for 30 min at 30 °C in Eppendorf Thermomixer Compact with continuous shaking at 1250 rpm. Enzymatic reactions were terminated by addition of 375 µL chloroform:methanol (1:2; *v*:*v*), 5 µL of glacial acetic acid, 125 µL of chloroform and 125 µL of water. The samples were mixed vigorously and centrifuged to separate bottom chloroform layer from upper methanol-water layer. Chloroform fractions containing lipids were collected and separated by thin-layer chromatography method on silica gel 60 plates from Merck using polar solvent system consisting of chloroform:methanol:acetic acid:water (90:15:10:2.5 or 90:17.5:10:3.5; *v*:*v*:*v*:*v*). [^14^C]PC products were visualized and measured by electronic autoradiography (Instant Imager, Packard Instrument Co., Downers Grove, IL, USA).

Additionally to the assays measuring substrate specificity toward acyl donors of the tested *Pt*LPCAT1, we performed assays to evaluate the corresponding substrate specificity of LPCATs of the microsomal fraction of developing *C. sativa* seeds (24 days after flowering). We used *sn*-1-[^14^C]18:1-LPC as acyl acceptor and several acyl-CoA with long and very long-chain polyunsaturated fatty acids as acyl donors. The assays conditions were as described above for yeast microsomal fraction with aliquots of microsomes containing 0.2 nmol microsomal PC/assay (for additional information, see Klińska et al. [11]).

## Data Availability

See supplementary materials, no other data available elsewhere.

## Supplementary Materials

The following are available online at https://www.mdpi.com/article/10.3390/ijms22169056/s1, Table S1: LPCAT activity of proteins encoded by six different genes cloned from *P. tricornutum* with a sequence similar to genes encoding acyl:CoA:lysophosphatidylcholine acyltransferases (LPCATs) in other organisms.
