# Supplementary material for: Diatoms and Plants Acyl-CoA:lysophosphatidylcholine Acyltransferases (LPCATs) Exhibit Diverse Substrate Specificity and Biochemical Properties
Source: Int J Mol Sci. 2021 Aug 22;22(16):9056. doi: 10.3390/ijms22169056 (PMC8396554; doi:10.3390/ijms22169056)
Supplement: Supplementary file 1 [file ijms-22-09056-s001.zip › ijms-1350255-supplementary.pdf]

Int. J. Mol. Sci.

Diatoms and plants acyl-CoA:lysophosphatidylcholine acyltransferases (LPCATs) exhibit diverse substrate specificity and biochemical properties

Ada Połońska, Katarzyna Jasieniecka-Gazarkiewicz, Lingjie You, Xiahui Hao, Sylwia Klińska, Gangmin Gong and Antoni Banaś

Supplementary materials

Table S1. LPCAT activity of proteins encoded by six different genes cloned from *P. tricornutum* with sequence similar to genes encoding acyl:CoA:lysophosphatidylcholine acyltransferases (LPCATs) in other organisms.

**Tested gene**	**pmol [^{14}C]PC/nmol microsomal PC/min**
**Phatr3_J54709**	**0.05 ±0**
**Phatr3_J11916**	**0.06±0.02**
**Phatr3_J40461**	**0.14±0.02**
**Phatr3_J43099**	**0**
**Phatr3_J20460**	**140.4±9.3**
**Phatr3_J48640**	**0**

Cloned genes were heterologously expressed in yeast lacking functional gene encoding ALE1 (acyl:CoA:lysophosphatidylcholine acyltransferase of yeast). In assays we used microsomal fractions from these yeast as an enzyme source and LPC as acyl acceptor in combination with [^{14}C]18:1-CoA as acyl donor. Values shown are differences of activities between microsomal fractions of yeast *Δale1* expressing *P. tricornutum* cloned genes and activities of control yeast (*Δale1*) harboring an empty vector. Mean values and SD are presented (data from at least two independent assays).
